# AFTER-CA: Autonomic Function Transformation and Evaluation Following Catheter Ablation in Atrial Fibrillation

**DOI:** 10.3390/jcm13195796

**Published:** 2024-09-28

**Authors:** Monica Ferreira, Pedro Silva Cunha, Ana Clara Felix, Helena Fonseca, Mario Oliveira, Sergio Laranjo, Isabel Rocha

**Affiliations:** 1 Faculdade de Medicina and Centro Cardiovascular da Universidade de Lisboa-CCUL, Universidade de Lisboa, 1649-004 Lisbon, Portugal; ferreira_t_monica@hotmail.com (M.F.); mmartinsoliveira@gmail.com (M.O.); 2 Cardiology Department, Hospital de Santa Marta, Unidade Local de Saúde de S. José, 1150-199 Lisbon, Portugal; psilvacunha@gmail.com (P.S.C.); helena.fonseca@ulssjose.min-saude.pt (H.F.); 3 Pediatric Cardiology Department, Hospital de Santa Marta, Unidade Local de Saúde de S. José, 1150-199 Lisbon, Portugal; ana.felix@ulssjose.min-saude.pt (A.C.F.); sergiolaranjo@gmail.com (S.L.); 4 CHRC, NOVA Medical School, Faculdade de Ciências Médicas, NMS, FCM, Universidade NOVA de Lisboa, 1169-056 Lisboa, Portugal

**Keywords:** atrial fibrillation, catheter ablation, autonomic function, heart rate variability, baroreflex sensitivity, pulmonary vein isolation, cryoablation, quality of life

## Abstract

**Background:** Catheter ablation (CA) is a well-established treatment for atrial fibrillation (AF). However, its effects on autonomic function and underlying mechanisms remain poorly understood. This study investigated autonomic and haemodynamic changes following CA and explored their potential implications for patient outcomes. **Methods**: Seventy-eight patients with AF underwent CA and were followed up at one, three, and six months. Autonomic function was assessed using a combination of head-up tilt (HUT), handgrip (HG), and deep breathing (DB) manoeuvres along with baroreflex sensitivity (BRS) and baroreflex effectiveness index (BEI) evaluation. Heart rate (HR), blood pressure (BP), and their variability were measured at each time point. **Results**: Significant autonomic alterations were observed after ablation, particularly at one month, with reductions in parasympathetic tone and baroreflex function. These changes gradually normalised by six months. Both pulmonary vein isolation (PVI) and cryoablation (CryO) had similar effects on autonomic regulation. Improvements in quality of life, measured by the AFEQT scores, were consistent with these physiological changes. **Conclusions:** CA for AF induces significant time-dependent autonomic and haemodynamic changes with recovery over six months. These findings underscore the need for ongoing monitoring and personalised post-ablation management. Further research is required to explore the mechanisms driving these alterations and their long-term impacts on patient outcomes.

## 1. Introduction

Atrial fibrillation (AF) is a common cardiac arrhythmia that poses significant clinical challenges due to its association with increased morbidity and mortality [1]. Characterised by irregular and often rapid atrial activity, AF is linked to a heightened risk of stroke, heart failure, and other cardiovascular complications [1,2,3]. Management of AF includes both pharmacological and procedural interventions aimed at restoring and maintaining sinus rhythm. Among these interventions, catheter ablation (CA) has become a well-established treatment, particularly in patients with symptomatic AF who are refractory to medication. This technique uses various energy sources, such as radiofrequency (RF), cryotherapy, and pulse field ablation, to isolate or eliminate electrical triggers responsible for AF [4,5,6,7,8].

Pulmonary vein isolation (PVI) is the most commonly employed target for ablation; however, alternative approaches, such as antral ablation and cardioneuroablation, have attracted increasing attention due to their potential effects on the autonomic nervous system (ANS) [9,10,11,12,13]. Antral ablation extends the ablation area to regions around the pulmonary veins, potentially influencing autonomic inputs to the heart by targeting ganglionated plexi (GP), which are key components of the ANS. Modifying these autonomic inputs may help reduce AF recurrence [14,15]. Similarly, cardioneuroablation, whether performed alone or alongside traditional ablation techniques, seeks to modulate autonomic pathways involved in AF, further emphasising the role of the ANS in AF pathophysiology [4,5,16,17,18,19].

The scientific rationale for investigating post-ablation autonomic function arises from growing evidence that highlights the pivotal role of the ANS in the development and maintenance of AF. Autonomic imbalances—characterised by increased sympathetic activity and diminished parasympathetic influence—have been identified as key contributors to AF through a process known as autonomic remodelling [20,21,22,23,24,25]. This remodelling, which involves structural and functional changes in the ANS, increases the heart’s susceptibility to arrhythmias. Additionally, research has shown that CA, particularly when it affects the ganglionated plexi near the ablation sites, can inadvertently modulate autonomic inputs, thereby contributing to reduced AF recurrence [26,27,28,29]. Therefore, understanding how CA influences autonomic tone has significant implications for improving long-term AF outcomes.

Previous studies have consistently reported changes in autonomic function following CA, with varying effects on AF recurrence. For example, ablation-induced modulation of parasympathetic and sympathetic tone has been linked to both transient reductions in arrhythmia burden and improved long-term outcomes [30,31,32,33]. These autonomic shifts are particularly relevant during the “blanking period”—a critical three-month post-ablation phase marked by transient arrhythmias, as the heart undergoes healing and remodelling [34]. Investigating how autonomic changes during this period impact long-term outcomes is crucial for refining ablation strategies and predicting AF recurrence.

We prioritised the hypothesis that autonomic tone changes in patients following CA, regardless of the specific technique used, and that these changes may play a crucial role in determining the likelihood of AF recurrence. The behaviour of the ANS during the blanking period is particularly significant, as transient arrhythmias during this time may offer insights into the heart’s remodelling process. While predicting AF recurrence is a key goal, our primary research question focuses on how post-ablation autonomic changes can serve as predictors of recurrence, potentially guiding more effective patient management.

Nevertheless, several gaps in our understanding of the relationship between CA and autonomic function remain. Specifically, it is unclear to what extent different ablation techniques, such as antral ablation and cardioneuroablation, influence autonomic tone and contribute to the success or failure of AF ablation. Additionally, further investigation is needed into how autonomic remodelling during the blanking period affects long-term outcomes.

## 2. Methods

This prospective observational study was conducted at a tertiary care centre. The study protocol was approved by the Ethics Committee of ULS de S. José (Code INV357/CES n° 1282/2022; approval date 11 January 2023) and all procedures were performed in accordance with the ethical standards of the Declaration of Helsinki. Informed consent was obtained from all the participants prior to their inclusion in the study.

### 2.1. Subjects

**Figure 1:**
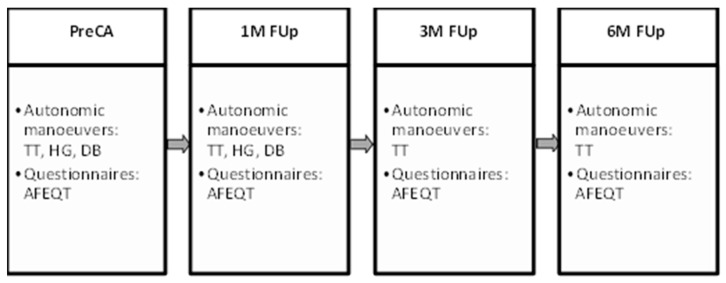
Protocol for autonomic and haemodynamic assessment of AF patients undergoing CA. This flowchart outlines the stepwise approach to autonomic and haemodynamic assessment performed before and after CA in AF patients. It highlights the key components of the assessment protocol, including the initial patient screening, baseline measurements of autonomic and haemodynamic parameters, the CA procedure, and subsequent follow-up assessments at 1-, 3-, and 6-month intervals after the procedure. These repeated assessments allow a thorough comparison of pre- and post-ablation status so that significant changes in autonomic tone and haemodynamic function after the procedure can be identified.

This study enrolled patients consecutively, who were diagnosed with atrial fibrillation (AF), irrespective of its classification (paroxysmal, persistent, or long-standing persistent), and who were in normal sinus rhythm at the time of the initial evaluation. The inclusion criteria were broad, without restrictions on sex, age, or the presence of comorbidities, to ensure a diverse patient cohort and enhance the generalisability of the findings. However, specific exclusion criteria were applied to ensure that the autonomic changes observed were primarily related to the catheter ablation (CA) procedure. Patients with pre-existing autonomic dysfunctions, such as neuropathies, Parkinson’s disease, diabetes with autonomic neuropathy, multiple system atrophy, or those taking medications known to affect autonomic function (e.g., beta-blockers, anticholinergics, and certain antihypertensives), were excluded from the study. Comprehensive medical histories were reviewed for each patient, and detailed physical examinations were performed to assess overall health status. Pre-procedural assessments were conducted prior to CA, and follow-up evaluations were scheduled at defined intervals to monitor changes in autonomic tone and their association with AF recurrence (Figure 1).

### 2.2. Pre-Ablation Assessment

Approximately one week before the ablation procedure, all participants underwent a detailed pre-ablation assessment focused on evaluating ANS function. The following autonomic tests were performed:

- Tilt Table Test (HUT): Patients rested supine on a tilt table for 15 min before being tilted to a 70-degree angle for 5 min. Continuous heart rate (HR) and blood pressure (BP) monitoring was performed throughout the test to assess cardiovascular responses to postural changes. This test primarily evaluates autonomic regulation of BP and HR during orthostatic stress;
- Deep Metronomic Breathing Test (DB): Patients performed controlled breathing at a rate of six breaths per minute (5 s inhalation, 5 s exhalation) for one minute while seated. HR variability during this test was analysed to assess the parasympathetic function, as this test is particularly sensitive to changes in vagal activity;
- Handgrip Test (HG): Patients were instructed to maintain a handgrip at 30% of their maximum voluntary contraction using a dynamometer for three minutes. BP and HR were monitored to evaluate sympathetic adrenergic function, reflecting the cardiovascular system response to sustained isometric stress.

These tests were conducted in a controlled environment to minimise external factors that could influence autonomic responses. The results provided a baseline autonomic profile for each patient, which was later compared with the post-ablation data to identify any significant changes due to the intervention.

### 2.3. Catheter Ablation Procedure

Catheter ablation procedures were performed according to established protocols. Pre-procedure assessments included either an angio-CT scan to evaluate the pulmonary vein (PV) anatomy or a transesophageal echocardiogram to assess cardiac function and detect any potential thrombi. On the day of the procedure, patients were sedated with propofol and alfentanil or received general anaesthesia, as determined by the clinical criteria. Femoral vein access was obtained under echocardiographic guidance and left atrial access was achieved through transseptal puncture using a single-puncture technique. Anticoagulation was maintained with heparin to maintain the activated clotting time (ACT) above 300 s, with ACT reassessed every 20–30 min.

Two ablation strategies were employed, which were chosen based on the operator’s clinical judgment and individual patient characteristics:

1. Radiofrequency Pulmonary Vein Isolation (RF-PVI): Point-by-point radiofrequency energy was applied to create lesions around the pulmonary vein antrum, isolating the PVs from the rest of the left atrium (LA). The procedure was guided using a 3D electroanatomic mapping system (Carto 3, Biosense Webster, Irvine, CA, USA) and ablation index-guided catheters (Thermocool Smarttouch™ SF catheter);
2. Cryoablation (CryO): Cryoablation involves the use of a balloon catheter (Arctic Front Advance Pro, Medtronic, Dublin, Ireland) to deliver extreme cold to the PVs, creating lesions through tissue freezing. This technique has particularly been employed in patients with paroxysmal AF. The cryoballoon was positioned at each PV ostium and cryothermal energy was applied to achieve PV isolation.

### 2.4. Post-Ablation Follow-Up Assessments

Patients were systematically followed up at 1, 3, and 6 months after the ablation procedure to monitor recovery and assess AF recurrence. The primary endpoint for AF recurrence-free survival was defined as the absence of atrial tachyarrhythmias lasting 30 s or longer after the 90-day blanking period, as well as the absence of sustained symptomatic episodes of rapid palpitations, the prescription of antiarrhythmic drugs (class I or III), or the need for repeat ablation.

After the ablation procedure, patients were discharged on antiarrhythmic drugs (AAD) at the operator’s discretion, in conjunction with oral anticoagulation. Patients were seen at routine follow-up visits 1–3 months after the procedure and every six months for the first two years, or earlier if symptoms occurred. At each follow-up visit, a standard 12-lead ECG was performed. Between 30 and 90 days post-ablation, patients were monitored with an external loop recorder (Spiderflash™ and Eventscope™ 3 analysis software, MicroPort CRM, Clamart, France). After the blanking period, 24 h Holter monitoring was performed at each outpatient visit.

In terms of medication management, AADs were continued for six months after ablation, with the exception of beta-blockers, which were continued if patients remained free from arrhythmia-related symptoms. Oral anticoagulation was reassessed after three months, based on the patient’s CHA2DS2-VASc score. Any clinical events occurring during the follow-up were also documented and evaluated.

Additionally, the following autonomic tests were performed during follow-up:

- **One-Month Follow-Up**: All pre-ablation autonomic tests (HUT, DB, HG) were repeated to evaluate early changes in autonomic tone and assess the initial impact of the ablation procedure on the autonomic nervous system (ANS);
- **Three-Month Follow-Up**: The HUT was repeated to monitor autonomic function during the “blanking period”, a critical time window where transient arrhythmias are common as the heart undergoes healing and remodelling;
- **Six-Month Follow-Up**: A final HUT was conducted to assess long-term autonomic changes and the potential for AF recurrence.

### 2.5. Data Acquisition, Pre-Processing, and Analysis

Data acquisition was carried out using a Task Force Monitor (CNS Systems), which provided continuous, non-invasive measurements of heart rate (HR) and blood pressure (BP) during all autonomic tests including the tilt table test, deep metronomic breathing test, and handgrip test. This system ensured precise and consistent data collection at each time point: before catheter ablation and during follow-up assessments at one, three, and six months post-ablation.

Once the data were collected, preprocessing was performed to prepare the signals for analysis. The first step involved identifying and excluding outliers using Tukey’s fences method, which defines outliers as values below Q1–1.5*IQR or above Q3+1.5*IQR. This step was crucial in preventing extreme values from skewing the results and ensuring that subsequent statistical analyses were robust. Following outlier management, the raw signal data were filtered to remove noise and artefacts. Signals were then resampled to maintain uniform time intervals, which is necessary for accurate analysis in both the time and frequency domains. In addition, an algorithm was applied to detect and correct ectopic beats and periods of signal loss. These anomalies were replaced with interpolated values derived from adjacent regular RR intervals to maintain the data integrity.

The FisioSinal algorithm, developed in Python, was employed for the analysis. The pre-processed data were analysed using the Hilbert–Huang transform (HHT), a method well suited for the nonlinear and nonstationary nature of HRV and BP signals [21,27]. The HHT process begins with empirical mode decomposition (EMD), which decomposes signals into intrinsic mode functions (IMFs). These IMFs capture the inherent oscillatory modes within the original signals, with each mode characterised by variable amplitude and frequency over time. A Hilbert spectral analysis was then applied to each IMF to extract instantaneous frequency data, providing detailed insights into the frequency components of the HR and BP signals over time. Finally, a time–frequency–energy spectrogram was generated to visually represent the distribution of energy across different frequency bands throughout the observation period.

### 2.6. Autonomic Nervous System Assessment

#### 2.6.1. Baroreflex Assessment

The baroreflex sensitivity (BRS) and baroreflex effectiveness index (BEI) were assessed using the sequence method described by Rienzo and co-workers [28]. In short, the sequence method involves identifying sequences of spontaneous increases or decreases in systolic blood pressure (sBP) that are followed by the corresponding lengthening or shortening of the RR intervals. This approach allows for the evaluation of the ability of the baroreflex to regulate the heart rate in response to changes in blood pressure. Specifically, the BRS measures the slope of the linear relationship between the changes in sBP and the corresponding RR intervals, representing the reflex gain. On the other hand, BEI quantifies the proportion of baroreflex sequences within a given period, providing an index of the effectiveness of the baroreflex in modulating heart rate on a beat-to-beat basis. These parameters were computed from continuous blood pressure and ECG data recorded during autonomic tests, providing a detailed analysis of the autonomic regulation of cardiovascular function [21,27].

#### 2.6.2. Autonomic Tests Analysis

The analysis of autonomic test data was structured to capture specific physiological responses across defined time periods. For the tilt test, the data were divided into four distinct periods: B1, representing the last two minutes of the resting phase as a baseline; TA1, the first two minutes after tilting up; TA2, the subsequent two minutes post-tilt; and B2, the first two minutes of recovery after tilt-down. Initial studies have indicated that significant changes in autonomic tone occur during the first and second minutes of orthostatic adaptation. Therefore, the focus of this analysis was on these early periods of intense adaptation, with the later phase grouped to enhance the statistical power and ensure more reliable data interpretation. This refined approach aimed to standardise the analysis, making it applicable to broader clinical trials [29].

For the handgrip (HG) test, analysis focused on the low-frequency (LF) band of diastolic blood pressure during the final two minutes of the resting phase (B1) and throughout each of the three minutes of sustained isometric contraction (HG1–HG3). The analysis concentrated on intervals showing the greatest variation between the maximum and minimum values compared to baseline, reflecting the cardiovascular response to isometric stress.

In the deep metronomic breathing (DB) test, data were analysed over two periods: the final two minutes of rest before the test (B1) and the one-minute deep breathing phase (DB). The focus was on the maximum-to-minimum heart rate variation (DB-HRmax to DB-HRmin), comparing these changes with baseline values to assess the parasympathetic function.

### 2.7. Quality of Life Assessment

Patients’ quality of life (QoL) was assessed using the Atrial Fibrillation Impact on Quality-of-Life Questionnaire (AFEQT) at baseline and during follow-ups at 1, 3, and 6 months post-ablation. The AFEQT is a validated tool designed to evaluate the impact of AF on health-related quality of life (HRQoL), as noted in prior research [30,31]. This self-administered questionnaire consists of 20 questions, with the first 18 addressing the impact of AF on daily life using a Likert scale (1–7) and the final two questions evaluating patient satisfaction with treatment, which did not contribute to the HRQoL score. The responses were converted into a 0–100 scale, where 0 represented severe symptoms or disability and 100 indicated no limitations. Higher scores reflect better health status. The total AFEQT score and its subscales were adjusted for missing responses using a standard formula: [100 − ((sum of severity scores for all questions answered − number of questions answered) × 100)/(total number of questions answered × 6)]. The validity of the Portuguese version of the AFEQT, as established by Darmits [32], supports its use in this study.

### 2.8. Statistical Analysis

Descriptive statistics, including mean values and standard errors of the mean (SEM), were calculated for each variable at all time points. Given the non-normal distribution of the data, non-parametric tests were employed throughout the analysis. The Wilcoxon signed-rank test was used to evaluate differences across the four time points (pre-ablation, one month, three months, and six months post-ablation) for each autonomic test. The Friedman test with Bonferroni correction was applied to compare the periods within each time point. This approach accounted for repeated measures and ensured the statistical robustness of the findings.

Bootstrap resampling was incorporated into the analysis to further enhance the reliability of the results. Additionally, potential confounders such as age, sex, baseline heart rate, blood pressure, and comorbidities were controlled to isolate the specific effects of catheter ablation on autonomic function. Bivariate correlations were examined using Pearson’s or Spearman’s correlation coefficients depending on the distribution of the variables in question. Multiple regression analysis was conducted to explore the predictive value of independent variables on outcomes, such as AF recurrence and autonomic changes. A *p*-value of less than 0.05 was considered indicative of statistical significance. All statistical analyses were performed using R (version 4.1.2) and R Studio (version 2021.09.1 + 372).

## 3. Results

This study included 78 patients, with a predominance of males (n = 50) and a mean age of 59 ± 10 years. Most patients were diagnosed with paroxysmal atrial fibrillation (PAF) (n = 67), while the remaining patients were diagnosed with persistent AF (n = 12). On average, patients presented with 2.5 comorbidities. Hypertension was the most common condition (n = 40), followed by diabetes (n = 8), obesity (n = 10), and smoking (n = 14). Obstructive sleep apnoea (OSA) was noted in two patients.

Regarding the ablation strategies used, 33 patients underwent RF point-by-point pulmonary vein isolation (PVI) and 45 were treated with cryoablation (CryO).

In terms of outcomes, the majority of the cohort (n = 72) did not experience recurrence of atrial fibrillation during the follow-up period, while a minority (n = 6) had recurrence. These findings suggest a favourable outcome for the majority of patients across the different ablation techniques employed.

### 3.1. Head-Up Tilt Assessment

#### 3.1.1. Haemodynamic Profile

**Figure 2:**
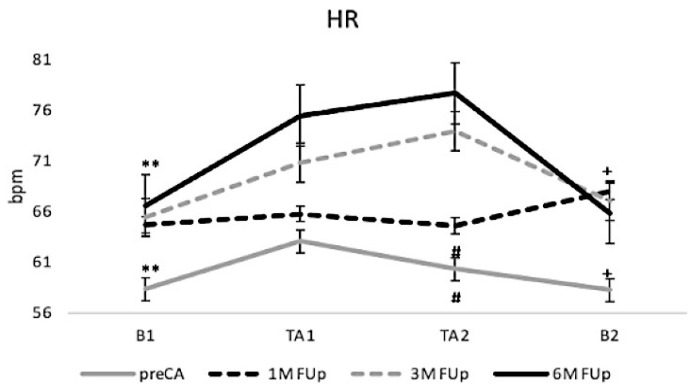
Temporal variation of HR in response to HUT. This figure shows the changes in HR observed at pre-CA and at 1M and 6M FUp. ** *p* < 0.001 at B1 between pre-CA and 1M FUp; ** *p* < 0.01 at B1 between pre-CA and 3M FUp; # *p* < 0.05 at B1 between pre-CA and 6M FUp; # *p* < 0.05 at TA2 between pre-CA and 1M FUp; ^+^ *p* < 0.01 at B2 between pre-CA and 1M FUp.

Over the course of the study, HR progressively increased from a baseline of 58 ± 1 bpm pre-CA to 67 ± 3 bpm at 6M FUp (*p* < 0.05) (gray line (pre-CA); Figure 2). This gradual increase suggests a shift from a cardiac stunning condition immediately after ablation to cardiac remodelling at the 6-month follow-up. During the tilt test, HR consistently rose across all time points, with the most pronounced increase at 6M FUp, where HR reached 77 ± 4 bpm at the tilt apex (TA2) (black line (6M FUp)). The steady rise from TA1 to TA2 across all follow-up periods, particularly in the 6M FUp group, likely reflects autonomic remodelling post-ablation, potentially indicating an increased parasympathetic sensitivity to the tilt challenge.

**Figure 3:**
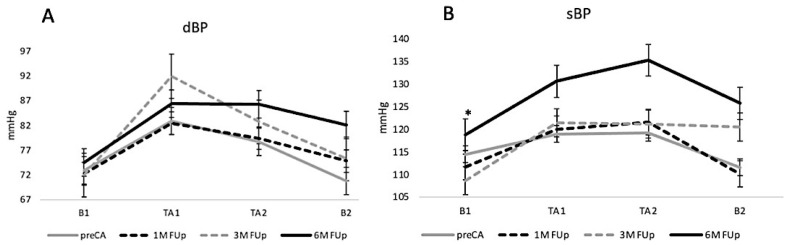
(**A**) Temporal variation of dBP in response to HUT from pre-CA until 6 months FUp. (**B**) Temporal variation of sBP in response to HUT from pre-CA until 6 months FUp; * *p* < 0.05 at B1 between pre-CA and 3M FUp.

Regarding BP, diastolic blood pressure (dBP) showed notable fluctuations despite being non-statistically significant, particularly during the tilt test, with a marked increase at the initial period of orthostatic stress at 3M FUp, which slightly stabilised by 6M FUp but remained above pre-CA levels (Figure 3A). Systolic blood pressure (sBP) demonstrated significant changes, with a pronounced increase observed during the tilt test at 3M FUp, peaking at TA2 in the 6M FUp group (Figure 3B). This increase suggests an adaptive cardiovascular response following ablation, with sBP stabilizing somewhat by 6M FUp, yet still showing differences compared to pre-ablation levels, indicating the longer-term physiological effects of the procedure.

**Figure 4:**
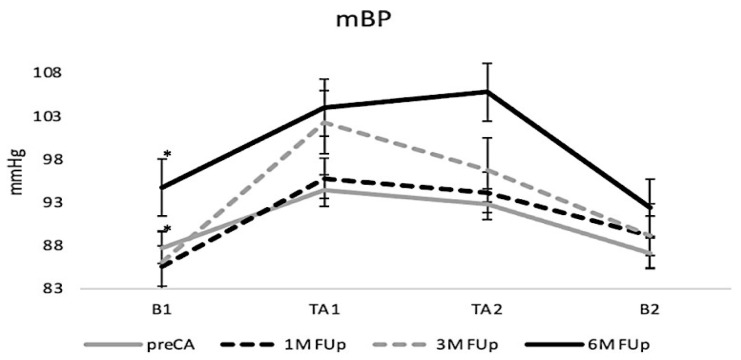
Changes in mean blood pressure (mBP) from pre-CA to 6M FUp. The figure illustrates the consistent increase in mBP from baseline to B2 at all measured moments; * *p* < 0.05 at B1 between pre-CA and 3M FUp.

The mean blood pressure (mBP) exhibited a progressive upward trend throughout the follow-up period (Figure 4). While there was a non-significant increase in mBP after one month, a statistically significant rise was observed at TA1 by the third month. By the sixth month, the mBP reached its peak, with significant increases recorded across all analysed periods (B1, TA1, TA2, and B2), indicating a continued upward trend and maximal values at 6M FUp (Figure 4).

**Figure 5:**
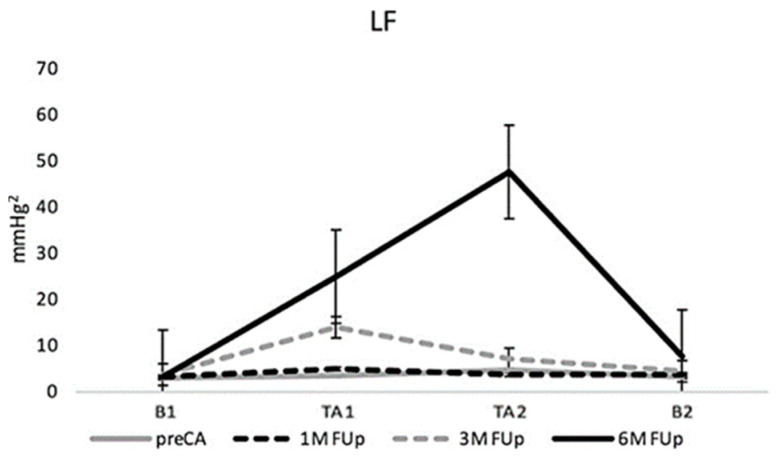
Longitudinal changes in LF band from pre-CA to 6M FUp, shedding light on the changes in sympathetic activity during the course of recovery from the ablation.

Autonomic responses to the HUT challenge showed significant changes from pre-CA to 6M FUp. Low-frequency (LF) band variability, associated with sympathetic activity, increased progressively throughout the follow-up period, reaching a significant peak at 6M FUp (Figure 5). This pronounced increase indicates a heightened sympathetic tone or stress response at 6M FUp. Following this peak, LF variability sharply declined, approaching baseline levels, suggesting a subsequent normalization of sympathetic activity.

#### 3.1.2. Variability Profile

Autonomic responses to the HUT challenge showed noticeable changes from pre-CA to 6M FUp. LF responses increase steadily during orthostasis and return to baseline during the tilt-down phase (B2). Over time, particularly at 6M FUp after catheter ablation, the LF responses become much more pronounced during the second phase of orthostasis, indicating heightened autonomic response compared to earlier stages.

**Figure 6:**
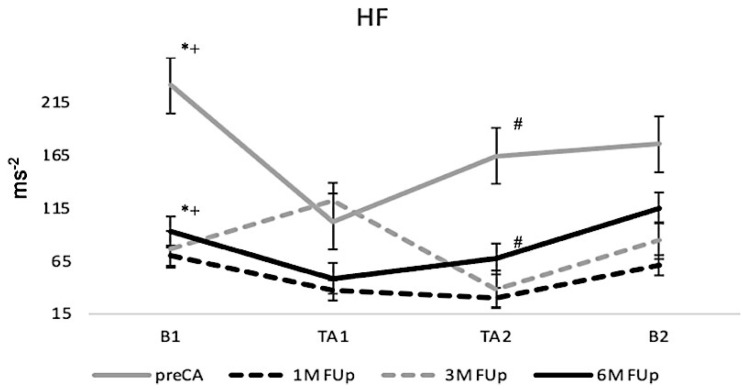
Evolution of HF band variability post-ablation. The figure displays the progression of HF band variability, a marker of parasympathetic activity, from pre-CA to 6M FUp. Notable changes in the HF band across distinct time points are showcased, providing insights into the parasympathetic modulation during the recovery process of FUp ablation. * *p* < 0.05 at B1 between pre-CA and 1M FUp; ^+^ *p* < 0.05 at B1 between pre-CA and 3M FUp; # *p* < 0.05 at TA2 between pre-CA and 3M FUp.

High-frequency (HF) band variability, reflecting parasympathetic activity, showed a marked decrease during the tilt test at 1M and 3M FUp compared to pre-CA (*p* < 0.05), suggesting reduced parasympathetic function post-ablation (Figure 6). Specifically, HF variability was markedly lower during the first and second tilt phases (TA1 and TA2) at these follow-ups, with statistical significance (*p* < 0.05). However, with 6M FUp, HF variability showed signs of recovery during the tilt phases, although it did not fully return to the pre-ablation levels. This indicates a potential long-term change in the parasympathetic activity following ablation.

#### 3.1.3. Baroreflex Function

**Figure 7:**
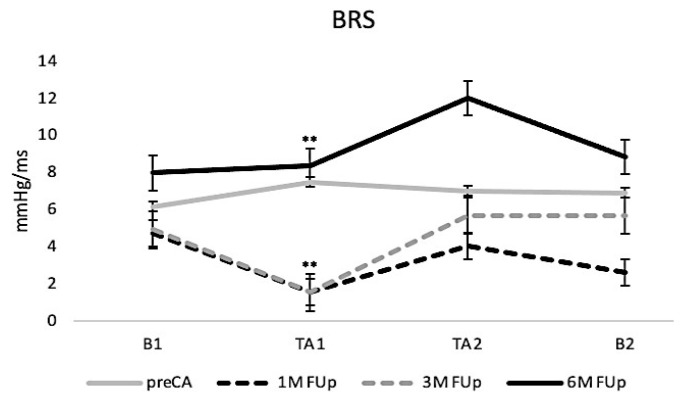
Baroreflex sensitivity (BRS) responses to TT. The lines represent the mean BRS at baseline, TA1 and TA2 during pre-ablation, and at 1, 3, and 6 months FUp. A significant decrease is observed at 1 month and 3 months FUp and an increase is observed at 6 months FUp; ** *p* < 0.01 at TA1 between pre-CA and 1M FUp.

Baroreflex sensitivity (BRS) was significantly altered after ablation (Figure 7). At 1M FUp, the BRS decreased significantly during the initial tilt phase (TA1), suggesting reduced baroreflex gain shortly after the procedure. However, with 3M FUp, BRS increased, particularly during the second tilt phase (TA2), indicating recovery, at least partial, of the baroreflex function. By 6M FUp, BRS values increased further, suggesting the stabilisation and potential normalisation of cardiovascular autonomic control.

**Figure 8:**
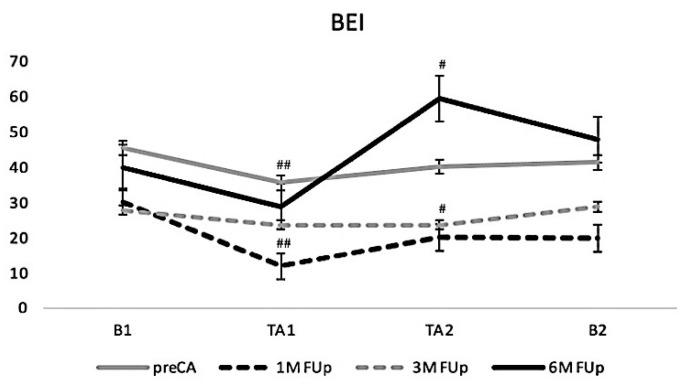
Baroreflex effectiveness index (BEI) responses to TT. The lines represent the mean BEI at baseline, TA1 and TA2 during pre-ablation, and at 1, 3, and 6 months FUp. A significant decrease is observed at 1 month and 3 months FUp and an increase is observed at 6 months FUp; # *p* < 0.01 at TA2 between pre-CA and 3M FUp; ## *p* < 0.01 at TA1 between pre-CA and 1M FUp.

The baroreflex effectiveness index (BEI) also exhibited dynamic changes following CA (Figure 8). At 1M FUp, the BEI decreased significantly during the initial phase of tilt TA1 and remained low during TA2 (*p* < 0.01), indicating an initial impairment in baroreflex function after CA. However, by 3M FUp, BEI values showed signs of recovery although they remained below pre-ablation levels, particularly during TA2, suggesting an adaptive improvement in baroreflex function over time. By 6M FUp, BEI values further increased especially during TA2, suggesting a near or full return to pre-ablation levels and reflecting the long-term effects of ablation on baroreflex efficacy.

### 3.2. Handgrip Manoeuvre

**Figure 9:**
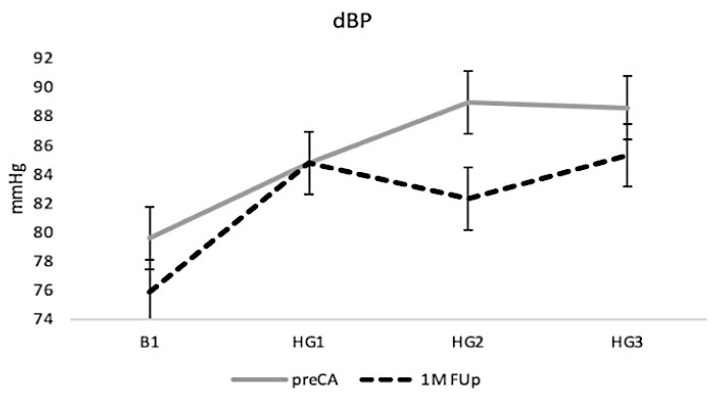
Diastolic blood pressure (dBP) responses to handgrip manoeuvre at pre-CA and 1M FUp. HG1–3 refers to the period of data analysis.

The handgrip (HG) manoeuvre demonstrated a typical physiological response with an increase in dBP due to increased peripheral resistance and sympathetic nervous system activity during muscular exertion (Figure 9). Before CA, dBP showed a steady rise throughout the HG manoeuvre, reflecting the body’s adaptation to physical stress by adjusting blood pressure to meet the increased workload demands. However, at 1M FUp, this response was notably blunted. The expected increase in dBP was less pronounced, starting from a higher baseline but showing a diminished rise during the handgrip exercise and particularly during the middle phase (HG2), where a dip in dBP was observed. This attenuated response suggests that CA may have altered the vascular or autonomic regulation of blood pressure, potentially indicating a change in the cardiovascular system response to stress after the procedure.

**Figure 10:**
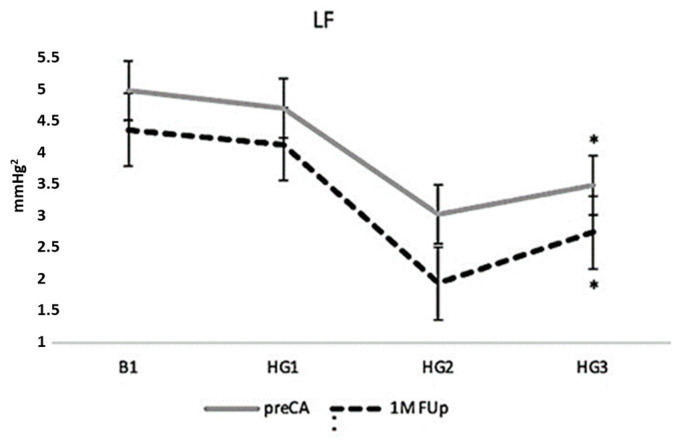
Low-frequency (LF) response to handgrip manoeuvre pre-CA and 1M FUp. The graph illustrates the mean LF values at baseline and along the manoeuvre; * *p* < 0.01 in HG3 between pre-CA and 1M FUp.

In terms of autonomic responses, the LF component, which primarily reflects sympathetic activity, showed a decreasing trend during the latter part of the HG manoeuvre. This trend persisted at 1M Fup, where LF values were significantly lower at the final stage of the HG test (HG3) than at pre-ablation (*p* < 0.01) (Figure 10). This reduction in LF, alongside the blunted dBP response, may suggest a shift in the balance between sympathetic and parasympathetic influences on cardiovascular function post-ablation, with a possible reduction in the sympathetic modulation of heart rate and blood pressure during physical stress.

### 3.3. Deep Breathing Manoeuvre

**Figure 11:**
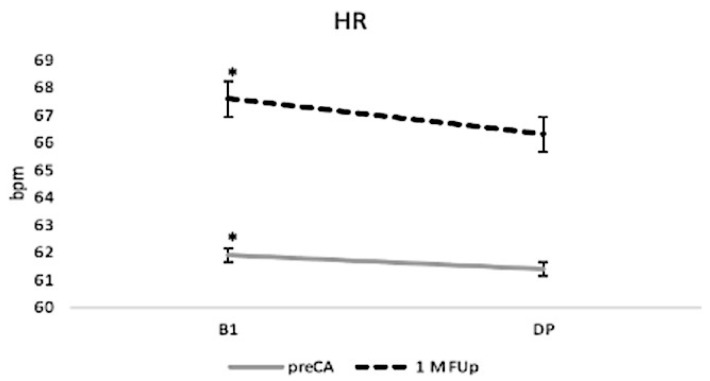
Changes in heart rate (HR) during DB pre-CA and at 1M FUp. The figure shows the mean HR values during the DB test, comparing the pre-ablation phase to the 1-month follow-up; * *p* < 0.05 at B1 between pre-CA and 1M FUp.

The deep breathing (DB) manoeuvre revealed distinct changes in both chronotropic and autonomic responses following catheter ablation (Figure 11). Baseline HR increased significantly from 62 ± 2 bpm pre-ablation to 67 ± 3 bpm at 1M FUp (*p* < 0.05). During the deep breathing (DP) phase, HR decreased slightly in both pre-CA and 1M FUp groups, but the HR remained consistently higher at 1M Fup compared to pre-CA. Although some variability was observed between different patient groups, such as normal-weight versus obese individuals, these differences did not reach statistical significance. HR fluctuations during the DB manoeuvre became more pronounced after ablation, although these changes were not statistically significant.

**Figure 12:**
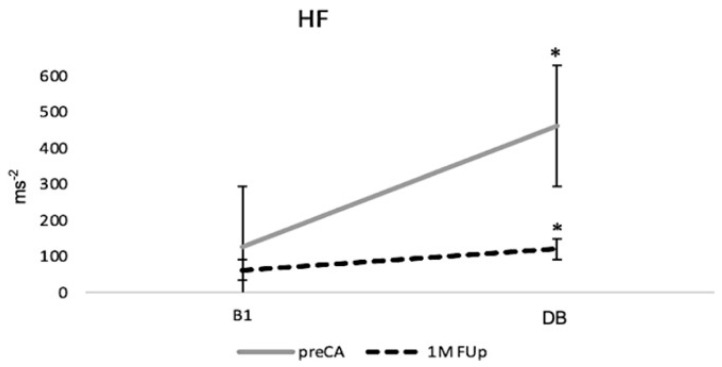
Changes in the high-frequency (HF) component of heart rate variability (HRV) before and after catheter ablation (CA); * *p* < 0.05 at DB between pre-CA and 1M FUp.

Regarding autonomic tone, the high-frequency (HF) component of heart rate variability (HRV), which is associated with parasympathetic activity, showed a marked increase during the DB manoeuvre before ablation (Figure 12). However, at 1M FUp, the HF values were significantly diminished both at rest and during the DB manoeuvre (*p* < 0.05), indicating a reduction in parasympathetic tone. This decrease in HF post-ablation suggests that the procedure may have affected the autonomic nervous system, particularly the vagal control of the heart rate, leading to a relative autonomic imbalance during the early recovery period.

These findings from the HG and DB manoeuvres highlight the potential impact of catheter ablation on both sympathetic and parasympathetic regulation, suggesting that the procedure may induce changes in autonomic function that persist into the early post-ablation period.

### 3.4. Catheter Ablation Effect on Quality of Life

**Figure 13:**
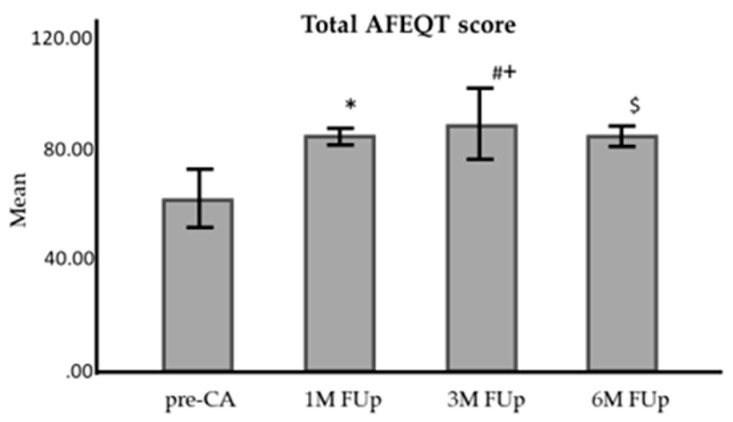
Comparison of total AFEQT questionnaire score before and at 1 month, 3 months, and 6 months FUp; AFEQT—atrial fibrillation effect on quality of life; pre-CA—before CA; 1M—one month; 3M—3 months; 6M—6 months. * *p* < 0.001 between pre-CA and 1M FUp; # *p* < 0.001 between pre-CA and 3M FUp; ^+^ *p* < 0.01 between 1M FUp and 3M FUp; ^$^ *p* < 0.01 between pre-CA and 6M FUp; error bars: 95%CI.

The AFEQT scores improved significantly after catheter ablation (CA). Before CA, the mean score was 54.5 ± 2.82, increasing to 78.9 ± 1.93 at one month (1M FUp), 84.7 ± 2.20 at three months (3M FUp), and stabilizing at 84.6 ± 4.04 at six months (6M FUp). The improvements were statistically significant from pre-CA to each follow-up (*p* < 0.001) and between 1M and 3M FUp (*p* < 0.01) (Figure 13).

**Figure 14:**
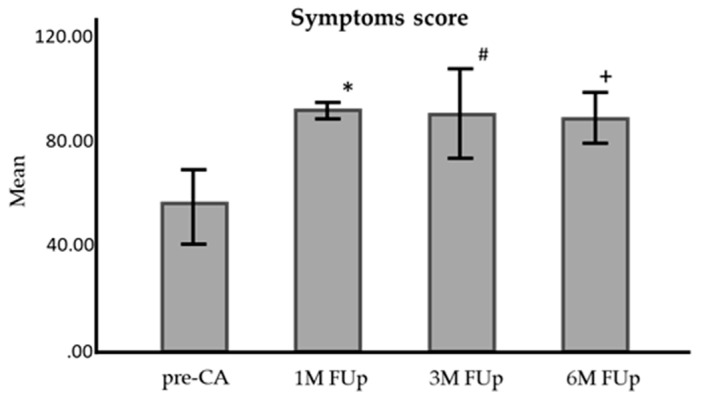
Comparison of symptoms score (q: 1–4) of AFEQT questionnaire before and at 1 month, 3 months, and 6 months FUp. AFEQT—atrial fibrillation effect on quality of life; pre-CA—before CA; 1M—one month; 3M—3 months; 6M—6 months; * *p* < 0.001 between pre-CA and 1M FUp; # *p* < 0.001 between pre-CA and 3M Fup; ^+^ *p* < 0.01 between pre-CA and 6M Fup; error bars: 95%CI.

The symptom score also showed significant improvement, rising from 50.99 ± 3.44 before CA to 82.1 ± 2.49 at 1M FUp, 83.6 ± 3.10 at 3M FUp, and 86.6 ± 4.03 at 6M FUp (*p* < 0.001 for 1M FUp, *p* < 0.01 for 3M and 6M FUp) (Figure 14).

**Figure 15:**
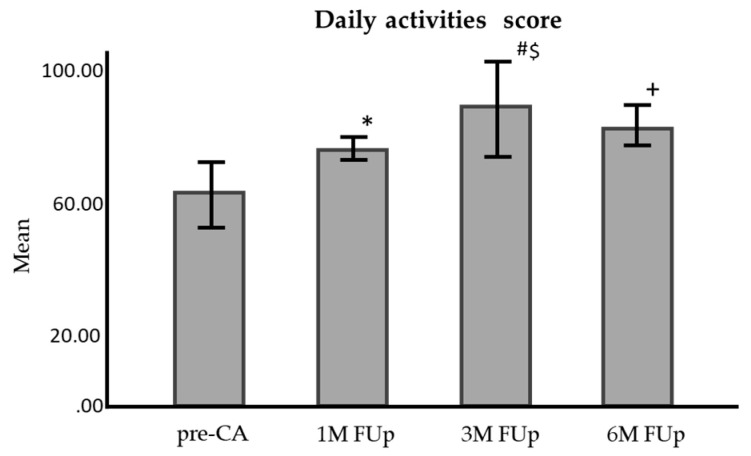
Comparison of daily activities questionnaire score before and at 1 month, 3 months, and 6 months FUp; AFEQT—atrial fibrillation effect on quality of life; pre-CA—before CA; 1M—one month; 3M—3 months; 6M—6 months; * *p* < 0.001 between pre-CA and 1M FUp; # *p* < 0.001 between pre-CA and 3M FUp; ^+^ *p* < 0.01 between pre-CA and 6M FUp; ^$^ *p* < 0.01 between 1M FUp and 3M FUp; error bars: 95%CI.

For daily activities, the score increased from 54.1 ± 3.30 before CA to 71.9 ± 2.28 at 1M Fup, 81.9 ± 2.57 at 3M Fup, and 82.6 ± 2.45 at 6M Fup, with significant differences at each follow-up (*p* < 0.001 for 1M Fup, *p* < 0.01 for 3M and 6M Fup) (Figure 15).

**Figure 16:**
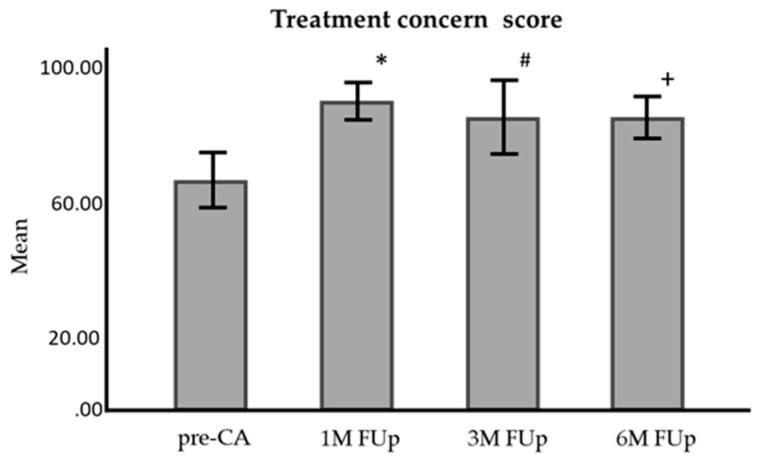
Comparison of treatment concern score of AFEQT questionnaire score before and at 1 month, 3 months, and 6 months FUp; AFEQT—atrial fibrillation effect on quality of life; pre-CA—before CA; 1M—one month; 3M—3 months; 6M—6 months; * *p* < 0.001 between pre-CA and 1M FUp; # *p* < 0.001 between pre-CA and 3M FUp; ^+^ *p* < 0.001 between pre-CA and 6M FUp; error bars: 95%CI.

Treatment concern scores improved from 61.2 ± 2.28 pre-CA to 86.0 ± 2.21 at 1M FUp, 85.5 ± 2.30 at 3M FUp, and 85.2 ± 2.74 at 6M FUp, with significant differences at all follow-ups (*p* < 0.001) Figure 16).

**Figure 17:**
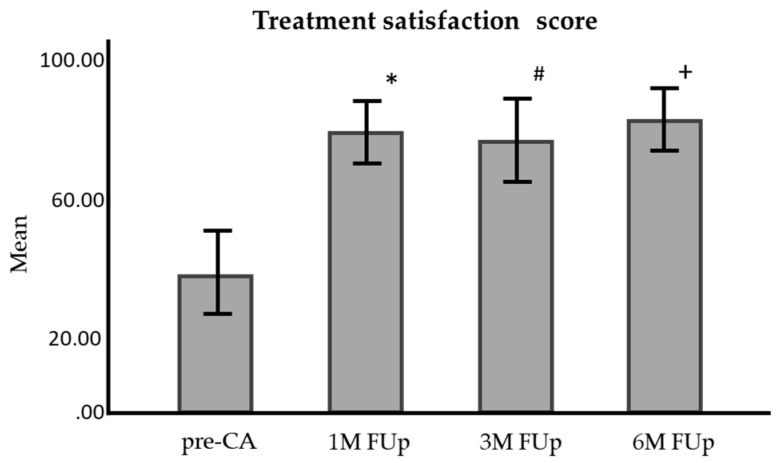
Comparison of treatment satisfaction questionnaire score before and at 1 month, 3 months, and 6 months FUp; AFEQT—atrial fibrillation effect on quality of life; pre-CA—before CA; 1M—one month; 3M—3 months; 6M—6 months; * *p* < 0.001 between pre-CA and 1M FUp; # *p* < 0.001 between pre-CA and 3M FUp; ^+^ *p* < 0.001 between pre-CA and 6M FUp.

Treatment satisfaction also increased from 43.6 ± 2.85 pre-CA to 86.0 ± 2.21 at 1M FUp. Despite a dip to 72.8 ± 3.04 at 3M FUp, the score rebounded to 81.5 ± 4.34 at 6M FUp, with all changes being statistically significant (*p* < 0.001) (Figure 17).

Overall, catheter ablation led to significant and sustained improvements in quality of life, with positive changes across all AFEQT domains from pre-ablation to follow-up, despite some variability in treatment satisfaction at 3M FUp.

## 4. Discussion

This study provides novel insights into time-dependent autonomic and haemodynamic changes following AF catheter ablation (CA). The key innovation of our research lies in the comprehensive analysis of autonomic responses through a combination of provocative manoeuvres, baroreflex assessments, and detailed follow-up over six months. Unlike many previous studies that primarily focused on heart rate variability (HRV) as a marker of autonomic function, our study expands the understanding of post-ablation recovery by integrating multiple autonomic tests, including head-up tilt (HUT), handgrip (HG), and deep breathing (DB) manoeuvres. This approach allows for a more nuanced understanding of how CA impacts both the sympathetic and parasympathetic systems over time.

Our findings demonstrated that CA induces significant and dynamic changes in autonomic function, particularly during the early post-ablation period. The observed alterations in heart rate (HR), blood pressure (BP), and autonomic tone suggest that CA disrupts cardiovascular homeostasis, leading to a temporary state of autonomic “stunning”. This is evidenced by the blunted responses in both HR and BP during the HG and DB manoeuvres at one month post-ablation, indicating reduced autonomic regulation during this early phase. Over time, these autonomic responses gradually recovered, as reflected in the increased baroreflex sensitivity and baroreflex effectiveness index (BEI) at six months post-ablation. The BEI, in particular, emerged as a valuable marker of autonomic recovery and could be integrated into routine post-ablation monitoring protocols to offer clinicians deeper insights into the recovery process and potentially predict long-term success.

Mechanistically, these findings can be attributed to the effect of CA on the intrinsic cardiac nervous system (ICNS). The ICNS plays a crucial role in regulating cardiac function, and its disruption through CA, particularly when involving pulmonary vein isolation (PVI) and ganglionated plexus (GP) ablation, likely contributes to the observed autonomic alterations. The temporary reduction in parasympathetic tone, as indicated by decreased high-frequency (HF) components and subsequent recovery, may reflect the process of autonomic remodelling, where the heart’s neural network gradually adjusts to the changes induced by ablation.

That said, it is important to consider that some of the autonomic changes we observed during the early post-ablation period could be partially influenced by transient post-procedural inflammation, rather than solely by long-term autonomic remodelling. Inflammation is known to disrupt autonomic regulation, leading to temporary changes in autonomic tone, especially in the immediate aftermath of CA. However, the gradual recovery of autonomic function, as evidenced by the improvements in BEI and parasympathetic tone over six months, suggests that long-term remodelling is a significant contributor. Future studies incorporating inflammatory biomarkers such as C-reactive protein or advanced imaging techniques like cardiac MRI could help differentiate between these two phenomena, providing a clearer understanding of the underlying mechanisms.

The haemodynamic responses observed during the HUT test further support the concept of autonomic remodelling. The initial increase in systolic and diastolic BP at one month post-ablation suggests a heightened sympathetic response, possibly as a compensatory mechanism for the loss of parasympathetic regulation. However, by six months, the BP responses appeared to stabilise, indicating a new equilibrium in cardiovascular control.

Our results are consistent with those of previous studies that reported similar autonomic changes following CA. For instance, Styczkiewicz and co-workers [33] found that the clinical success of PVI in preventing AF recurrence over six months was related to the degree of transient parasympathetic denervation, as reflected by a reduction in the BRS one month after the procedure. Similarly, Miyoshi and colleagues [34] demonstrated that antral PVI by radiofrequency (RF) depressed the BRS in patients with paroxysmal AF, suggesting transient parasympathetic suppression shortly after CA. These studies corroborate our findings, particularly the observed depression of the BRS and HF components at one month post-ablation and the subsequent recovery by six months.

Moreover, improvements in quality of life, as measured by the AFEQT questionnaire, further reinforce the positive effects of CA on patient outcomes. The significant enhancement in AFEQT scores from pre-ablation to follow-ups at one, three, and six months reflects the beneficial impact of autonomic and haemodynamic stabilisation on daily activities, symptom burden, and treatment satisfaction. These improvements are consistent with those of previous studies [30,35,36,37,38] that also demonstrated substantial quality-of-life gains following CA.

In contrast to some previous studies [39,40,41], our study did not find significant differences between patients who underwent RF or cryoablation in terms of autonomic recovery. This suggests that both techniques may exert similar neuromodulatory effects, likely because of the overlap between the ablation sites and GP locations. The work of Malcolme-Lawes and colleagues [18] supports this view by showing that PVI can disrupt neural connections to the pulmonary veins, leading to reduced autonomic regulation. Additionally, studies by Katritsis et al. [3] and Oswald et al. [17] have demonstrated that both RF and cryoablation modulate the ICNS, albeit temporarily, which aligns with our findings of autonomic changes up to six months post-ablation. The fact that newer techniques such as pulsed electric field ablation (PFA), which preferentially affects myocardial cells and spares neural tissue, have been shown to induce less prominent alteration of the cardiac ANS compared to RF ablation [16,42], supports the notion that the neuromodulatory effects observed with PVI and cryoablation are not merely incidental but are indeed a significant component of their therapeutic efficacy.

While our study focused on RF and cryoablation, recent evidence from studies on PFA suggests that the blanking period following ablation may vary depending on the energy source used. In particular, the work by Mohanty et al. (2024) demonstrated that early recurrence during the second and third months after PFA was predictive of late recurrence, leading the authors to suggest redefining the traditional three-month blanking period for PFA to one month. This raises important considerations for future studies, as different energy sources may induce distinct autonomic and inflammatory responses, thereby influencing the duration and nature of the blanking period. Although PFA was not used in our study, these findings could have implications for how autonomic recovery is interpreted across different ablation modalities [43].

The observed autonomic alterations highlight the importance of ongoing post-ablation patient monitoring. Given the dynamic nature of these changes, clinicians should be aware of the potential for transient autonomic dysfunction, particularly within the first three months following CA. This period may require more intensive follow-up and individualised care to ensure optimal recovery and minimise the risk of AF recurrence. Autonomic evaluation should encompass a comprehensive assessment of the autonomic reflex arc, including both sympathetic and parasympathetic branches. To achieve this, a combination of autonomic manoeuvres should be used: one that primarily evaluates mixed autonomic function, another focusing predominantly on sympathetic activity, and a third on parasympathetic function. Based on our findings, integrating the BEI into post-ablation follow-up protocols could provide clinicians with valuable insights into autonomic recovery and help guide treatment decisions.

This study adds to the growing body of literature on the autonomic effects of AF catheter ablation by providing a comprehensive time-dependent analysis of autonomic and haemodynamic responses. Our findings underscore the complex interplay between the cardiovascular and autonomic systems post-ablation and suggest that both RF and cryoablation have significant neuromodulatory effects. Future research should continue to explore the mechanisms underlying these changes and their long-term implications on patient outcomes.

### 4.1. Limitations

The small sample size of our study limited the statistical power and generalisability of our findings, particularly given the variability among patients with AF. Our cohort may not fully represent the broader AF population, especially with respect to different AF subtypes and the underrepresentation of female patients. Comorbidities, such as hypertension, which were common in our cohort, may also have influenced the results, potentially confounding the observed autonomic changes.

Additionally, while we focused on RF pulmonary vein isolation and cryoablation, these techniques may not fully capture the autonomic alterations associated with other ablation methods, such as pulsed field ablation. The six-month follow-up period may also be insufficient to fully reflect the long-term effects of catheter ablation on autonomic function, as autonomic remodelling may continue beyond this timeframe. Although we utilised a broad range of autonomic assessments, these may not encompass all facets of autonomic regulation, and factors such as patient compliance with testing could have influenced the accuracy of our measurements.

### 4.2. Future Directions

Future research should focus on larger and more diverse patient populations, including a wider representation of AF subtypes and female patients, to enhance the generalisability of the findings. Including control groups of AF patients who do not undergo ablation would also help to better isolate the effects of the ablation procedure on autonomic function and clinical outcomes.

Moreover, we are continuing the serial follow-up of our current cohort and expect to have robust long-term data in the near future. This extended follow-up will allow us to present medium- to long-term autonomic data, stratified by ablation type and other potential confounders. With this approach, we aim to provide deeper insights into whether the autonomic changes observed stabilise or evolve further over time, and how these changes correlate with long-term AF suppression and clinical outcomes.

In addition, further studies should focus on exploring the underlying mechanisms of autonomic changes after ablation, particularly the role of autonomic remodelling. Understanding these mechanisms could help guide patient management, with an emphasis on predicting AF recurrence and optimising recovery strategies. Ultimately, integrating autonomic assessments into routine post-ablation care may help refine personalised treatment approaches and improve patient outcomes.

## 5. Conclusions

Our study revealed significant autonomic alterations following catheter ablation for atrial fibrillation, driven by processes such as denervation, stunning, inflammation, and long-term remodelling. These changes, which are influenced by the type of ablation technique employed, may be associated with improved procedural outcomes and a reduced risk of AF recurrence. By using a range of autonomic assessments, including baroreflex evaluation, we were able to obtain a detailed understanding of these time-dependent changes and their impact on cardiovascular function.

The clinical implications of these findings suggest that post-ablation management could greatly benefit from a more tailored and dynamic approach. Routine follow-up after ablation should not only rely on traditional clinical evaluations but also include more detailed autonomic assessments, such as baroreflex sensitivity (BRS) and the baroreflex effectiveness index (BEI). These tools provide a more nuanced understanding of autonomic recovery and may serve as valuable indicators for predicting patient outcomes and guiding the approach to follow-up care. Incorporating these assessments into routine practice would allow clinicians to better identify patients at higher risk of recurrence and adjust their post-ablation monitoring protocols accordingly.

In addition, our findings raise the possibility that pharmacological management following catheter ablation should be adapted based on the patient’s autonomic recovery profile. Beta-blockers and certain antiarrhythmic drugs, particularly those in classes I and III, have the potential to interfere with the natural recovery of autonomic function. Therefore, clinicians should carefully evaluate the continued use of these medications during the post-ablation period. In patients who show positive signs of autonomic recovery, the gradual tapering or discontinuation of beta-blockers or antiarrhythmic drugs may promote a more natural restoration of autonomic balance. However, further research is necessary to determine the optimal timing for these adjustments and to assess their long-term impact on patient outcomes.

Given the complexity of autonomic recovery after ablation, a multidisciplinary approach to patient care may further improve outcomes. Integrating specialists in autonomic nervous system (ANS) function into arrhythmology teams would facilitate a more personalised management strategy, ensuring that autonomic changes are monitored closely and managed effectively. Such an approach could also help refine the use of pharmacological therapies, minimising their potential interference with autonomic recovery while maximising the benefits of ablation.

In conclusion, our study underscores the importance of adopting a personalised and dynamic approach to post-ablation management, with a particular focus on autonomic recovery. While our findings provide valuable insights into the time-dependent nature of autonomic changes following ablation, further research is needed to fully understand the underlying mechanisms driving these alterations. Future studies should also explore the long-term effects of pharmacological management on autonomic remodelling and refine patient-specific follow-up protocols to enhance procedural success and optimise recovery.

## Data Availability

Data are unavailable due to patient privacy reasons.
